# Enhancing learning experiences in pre-clinical restorative dentistry: the impact of virtual reality haptic simulators

**DOI:** 10.1186/s12909-023-04904-y

**Published:** 2023-12-12

**Authors:** Alaa Daud, Manal Matoug-Elwerfelli, Hanin Daas, Daniel Zahra, Kamran Ali

**Affiliations:** 1 Restorative Dentistry, College of Dental Medicine, QU Health, Qatar University, Doha, Qatar; 2 Dental Laboratories, College of Dental Medicine, QU Health, Qatar University, Doha, Qatar; 3 Assessment and Psychometrics, University of Plymouth, Peninsula Medical School, Faculty of Health, Plymouth, UK; 4 Oral Surgery, Associate Dean Academic Affairs, College of Dental Medicine, QU Health, Qatar University, Doha, Qatar

**Keywords:** Virtual reality, Haptic, Dental education, Curriculum, Dental students, Psychomotor skill

## Abstract

**Background:**

Utilization of Virtual Reality haptic simulation (VRHS) to aid in the training of various pre-clinical skills is of recent interest. The aim of this study was to evaluate the impact of VRHS in restorative dentistry on the learning experiences and perceptions of dental students.

**Methods:**

An interventional study design was utilized to recruit third year students. All participants provided informed consents and were randomly divided into two groups. Group 1: Initially performed a Class I cavity preparation with the VRHS, followed by the same exercise using the phantom head/ acrylic typodont teeth in a conventional simulation environment (CSE). Group 2: Initially performed Class I preparations in a CSE, followed by the same exercise using VRHS. Both groups performed the exercises on a lower right first molar. To understand students’ perception, an online questionnaire was circulated. Data analysis involved Chi-square tests, independent t-tests and Mann–Whitney U-tests using the R statistical environment package.

**Results:**

A total of 23 dental students participated in this study. Although student’s perceptions were similar in both groups, a strong agreement that VRHS training might be used to supplement standard pre-clinical training was noted. Advancements to the VRHS hardware and software are required to bridge the gap and provide a smooth transition to clinics.

**Conclusion:**

Novice dental students generally perceived VRHS as a useful tool for enhancing their manual dexterity. Dental institutions should endorse virtual reality technology with caution, ensuring a planned integration into the curriculum to optimize benefit. Feedback is pivotal to effective learning in simulation-based education, and the triangulation of feedback could serve as a powerful aid to maximize the learning experience.

## Introduction

Virtual reality (VR) entails the creation of virtual environments that enable interaction with users. Over the last decade, VR-based training is being used increasingly in healthcare education and appears to have a positive impact on supporting the development of clinical competence of students and trainees at the undergraduate and postgraduate levels [1, 2]. VR-based training has been used for learning a wide range of skills including medical history taking, clinical assessment, applied surgical anatomy, diagnostic procedures, and operative interventions [1]. Similarly, there is a growing trend of using Virtual Reality with haptic simulation (VRHS) to support the learning of undergraduate dental students in a variety of pre-clinical skills including but not limited to restorative dentistry, endodontics, prosthodontics, oral surgery, local anesthesia, and periodontology, to name a few [2]. Evidence from the existing literature suggests that VRHS in dental education has been largely used to supplement conventional training on dental models fixed in mannequins to simulate clinical settings [3]. However, further developments in VHRS may potentially allow it to replace conventional teaching on dental models.

Pre-clinical training using VRHS offers several advantages and can be used effectively to enhance core capabilities required for developing psychomotor skills such as coordination of hand-eye movement and consolidating fine motor skills [4]. Compared to physical models, VRHS offers a safer workspace for novice students and allows them to practice core skills repeatedly without the fear of damaging the teeth in physical models. Also, VRHS minimizes the risk of physical injuries from sharps such as burs, needles, and other sharp instruments [5]. VRHS also allows visualization of anatomical structures during operative procedures such as allowing the learners to evaluate the depth of drilling in the tooth and picture the underlying anatomical structures during surgical procedures [6]. Contemporary VRHS provide automated feedback on learner performance with reduced reliance on supervisor feedback, permitting students to learn core skills with limited supervision and build their confidence [7]. Haptic feedback during the use of drills, and other instruments also simulates real-life experience, though this element warrants further improvements [8]. Current VRHS technologies integrate an immersive virtual 3D experience into the simulation, creating the perception of touch/sensory feedback. The VRHS software displays the completion percentage, remaining caries, and over-drilled areas. This “real-time” feedback allows independent practice and enables educators to assess students’ performance fairly, in a standardized manner [9, 10]. Finally, minimal waste is produced during training on VRHS and despite the initial investment in the equipment, the running costs of VRHS is largely restricted to maintenance and costs for addition new procedures to the VRHS library.

A recent generation of dental VRHS devices has been introduced, some with advanced features, and others with minimal software development. Various forms of haptic feedback exist, including cutaneous feedback, which relates to pressure, shear, and vibrations applied to the skin, as well as kinesthetic feedback, which encompasses the forces and motion perceived by the muscles, tendons, and joints. Haptics have ushered in the use of mixed reality (MR) as an enhanced technology for dental training. Within MR, a fusion of virtual reality (VR) with the real world occurs, enabling the adjustment of VR volumetric models to engage with actual instruments while maintaining immersion in the physical environment [11]. Each VRHS differs in its fidelity and specific features, and most of them have been investigated thoroughly in the literature [12].

Notwithstanding the numerous advantages of VRHS, the existing equipment models warrant further developments in hardware and software to improve the ergonomic design, quality of haptic feedback, and the range of skills that can be practiced [13]. The VRHS Feedback from dental students suggests that a combination of traditional models in mannequins VRHS is the preferred approach and it is unlikely that VRHS would completely replace mannequins and models for the foreseeable future [14].

One of the core applications of VRHS in undergraduate dental education is related to caries removal and cavity preparation [15]. Currently, most dental schools use tooth models to train students on caries removal and cavity preparations. These are considered as fundamental skills for undergraduate dental students and provide them the foundations to apply them to other common dental procedures such as endodontic access, veneers, crown preparations, and tooth preparations for removable prosthesis [16]. Initial training of novice students on models is challenging due to the small size of teeth and potential risks of irreversible tooth damage and physical injuries associated with the use of handpiece and burs [17]. VRHS offers a safe learning environment for beginners and allows them to appreciate tooth anatomy, undertake caries removal, and prepare an appropriate cavity design before they consolidate these skills on physical models [18].

The aim of this study was to evaluate the impact of VRHS in restorative dentistry on the perceptions and experiences of undergraduate dental students and how it translates into development of skills in cavity preparations for simple resin composite restorations.

## Materials and methods

### Ethical approval

This study was an exploratory study to assess students’ perception of virtual reality haptics simulators (VRHS) on the student learning experience in preparation of a standard Class I cavity preparation for resin composite restorations. Ethical approval was obtained from the Institutional Review Board, Qatar University (QU-IRB 1652-EA/22).

### Setting

Qatar University, College of Dental Medicine

### Sampling technique and participants

A homogenous purposive sampling technique was performed to recruit all third-year dental undergraduate students (*n* = 23) at College of Dental Medicine, Qatar University. Email invitations to participate in the research were sent to all participants and included a participant information leaflet explaining the aim and objectives of the study. Participation was voluntary and all collected data was anonymized. A signed informed consent was obtained prior to commencing this study and students were assured that if they opt out of the study, this will not negatively affect their grades or course of study.

### Study design

#### Interventional study

For the purpose of this study, all 23 participants (mean age 22.3 years) were randomly divided into two groups through an online randomizing tool (www.randomizer.org. accessed on 5^th^ of September 2022). Both groups had to perform allocated tasks sequentially during scheduled sessions and had one tooth each (Table 1). The data collection was achieved within a two-week period, leading to the same endpoint:

**Table 1:** Allocated tasks and sequence

Group 1 (Study group)	Group 2 (Control group)
Natural tooth- Class I cavity preparation LR6^a^	Natural tooth- Class I cavity preparation LR6
**VR**^**b**^—Class I cavity preparation LR6	Acrylic tooth- Class I cavity preparation LR6
Acrylic tooth- Class I cavity preparation LR6	**VR**—Class I cavity preparation LR6

^a^LR6 = Lower right first permanent molar

^b^VR = Virtual reality

Group 1 (Study group): Initially trained to perform a Class I cavity preparation through the VRHS on a lower right first molar (tooth LR6), followed by the same exercise using the phantom head and acrylic typodont teeth in a conventional simulation environment (CSE).

Group 2 (Control group): Initially trained to perform Class I preparations in a CSE on the LR6, followed by the same exercise using VRHS.

### Virtual reality haptic simulator

In year 2 (previous to this study), students were trained on using the VRHS for the purpose of learning dental charting. In addition, students performed a manual dexterity exercise on a virtual block, following a stained “cross” shape. The aim of this exercise was to familiarize students with the grasp of the dental handpiece and the feedback sensation from the VRHS. The restorative course in year 3 is a combined theory and practical course, with the practical component taught in the simulation laboratory, using mannequin‐based phantom head and acrylic typodont teeth (Frasaco, ANKA-4 Z, Tettnang, Germany). Prior to commencing this study, year 3 participants received various sessions on caries investigation and diagnosis, rubber dam application (moisture control), instrumentation, principles of cavity preparation, and fissure sealant. As part of this study, participants also completed an occlusal cavity preparation (Class I) on mounted natural extracted human tooth. Natural teeth were carefully selected to be identical, and groups were homogeneous. The aim of practicing on natural teeth first was to allow students to experience the different sensory feedback and relate to the pressure required to cut in enamel and dentin, then transfer these skills to typodont (Frasaco) teeth and relate to the VRHS experience. All students received a step-by-step demonstration/guide on how to gain access to a carious lesion, remove caries bulk with a high-speed handpiece and remove remaining caries where necessary with a low-speed handpiece.

**Fig. 1:**
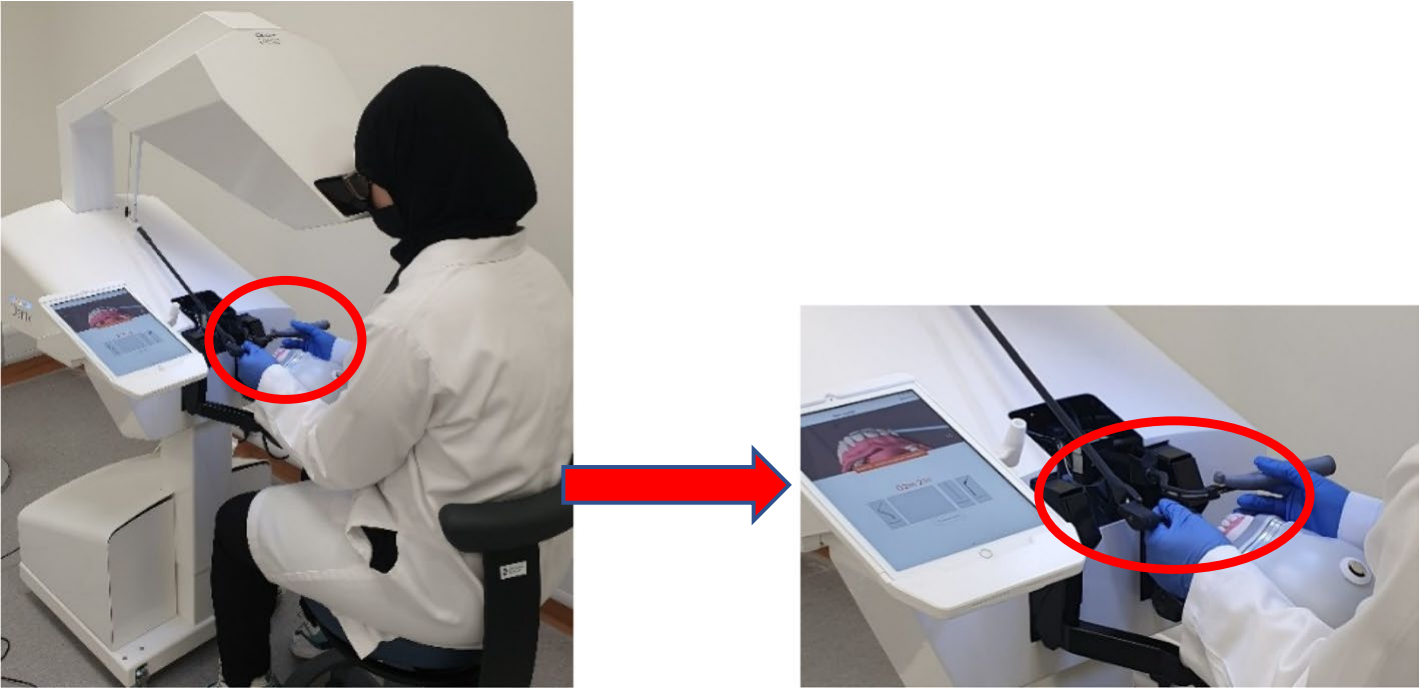
Virtual reality haptic simulator and handpiece

**Fig. 2:**
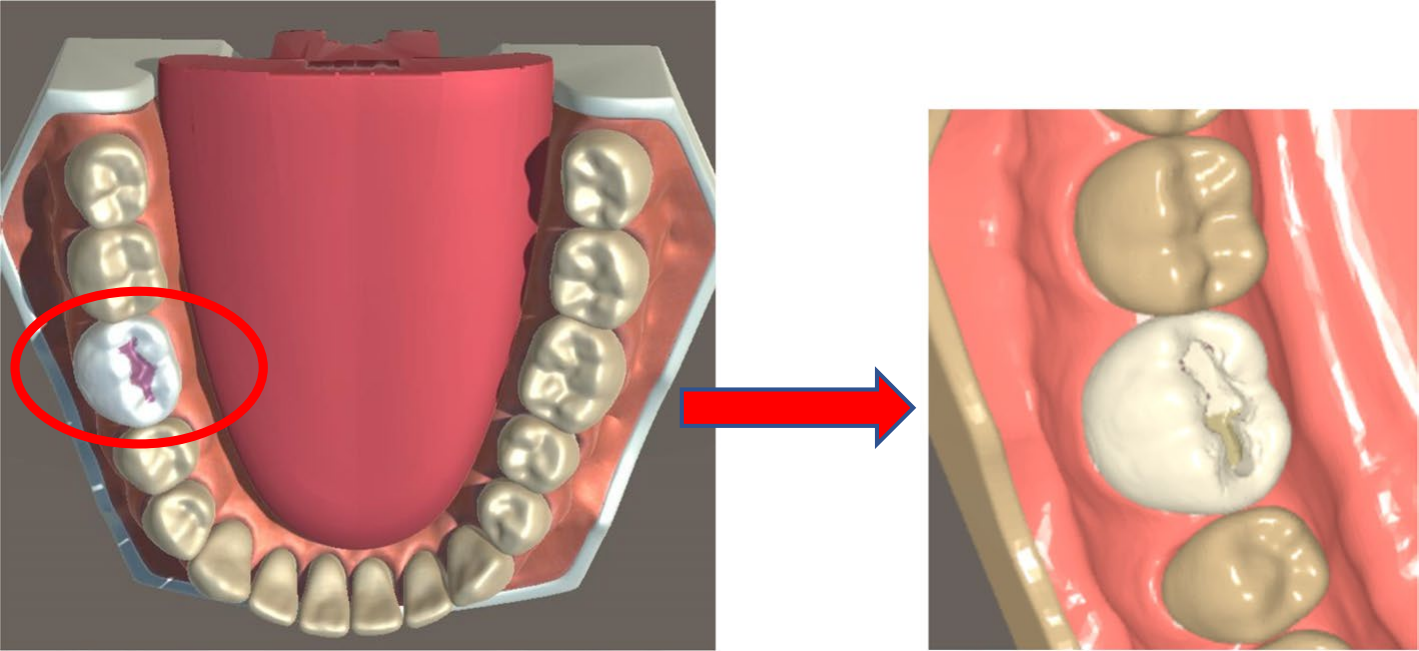
Caries removal exercise on virtual lower right first permanent molar tooth in the virtual reality haptic simulator

The VRHS hardware used (SIMtoCARE Dente®, SD001, Vreeland, The Netherlands) offers haptic force feedback while the user is performing virtual drilling. The interaction between the stylus (virtual handpiece) and the object (virtual lower first molar tooth; #36) produces visual changes (caries removal) in the 3D image of the #36, that is being displayed on the screen (Fig. 1). Specific dimensions and depth of occlusal caries were introduced by the supervisors (Fig. 2), and the texture (feedback sensation) of virtual caries was predetermined to differ from intact enamel and feel as close as possible to removing real caries. For each student, the simulator records the time spent, percentage of structure removed and any deviation from the allocated task. The software also enables students to repeat the task (reset the program) to improve their caries removal skills (Class I preparation). The virtual drilling takes place in a simulated phantom head (Frasaco, P-6/3, Tettnang, Germany) as part of the VRHS to mimic the head of a patient, enabling students to achieve appropriate finger rest while drilling. A foot pedal was used to control the speed of the handpiece. A mirror was also available for visualization and cheek retraction. Students were allocated 40 min each to accomplish and submit the task. Task performance was saved on a central server and each student had the chance to add comments regarding their experience.

### Dental mannequin with jaw models and teeth

For caries removal task on a mannequin, artificial teeth by Frasaco® (Frasaco dental model, ANKA-4 V CER, Tettnang, Germany) in the phantom head (similar to the phantom head used in VRHS) were used. The task involved caries removal mirroring the task on VRHS (2mm depth and 2mm width) in a #36 tooth with caries outlined on the occlusal surface of the tooth. Once students completed their tasks within the allocated time (40 min), feedback by calibrated, experienced supervisors were provided (blinded to study vs control group), and the first cavity preparation in the CSE was graded digitally on Blackboard (Blackboard Inc, US) The assessment rubric used a three-point scale (satisfactory, borderline, unsatisfactory) to evaluate cavity outline, depth, and efficacy of caries removal. The rubric was shared with the students on blackboard before the commencement of training and assessments.

### Data collection

On completion of all 3 tasks, an online questionnaire consisting of 14 closed-ended questions based on a Likert scale consisting of five categories: Strongly disagree, Disagree, Unsure, Agree, and Strongly agree. Data collection was achieved online using google forms. The questionnaire was adapted from Philip et al., 2023 and modified to fit the purpose of this study following piloting [19].

To further evaluate student perceptions and their experience related to VRHS, three open-ended questions were used. The questions investigated benefits and enablers, limitations and barriers, and recommendations to improve the student learning experiences in restorative dentistry with relation to VRHS.

### Data analysis

Analyses were conducted using the R statistical environment (R Core Team, 2022) [20]. Chi-square tests of association were conducted to compare the distribution of Strongly Agree, Agree, Neutral, Disagree, and Strongly Disagree responses for each Item between each group. Where a response was not used for either group, it was omitted from the contingency tables, and to account for some of the response options seldom being used for some items, the Chi-squared statistics were computed using Monte Carlo simulation with 10,000 replicates. This approach avoids making any assumptions about the nature of the Likert Scale data and reduces to impact of small counts within some categories.

Agreement responses for each Item were also subject to independent *t*-tests and Mann–Whitney *U*-tests, comparing the mean agreement scores between groups. These analyses were based on recoding of the agreement data to Strongly Agree = 2, Agree = 1, Neutral = 0, Disagree = -1, and Strongly Disagree = -2, and treating the data as continuous in the case of *t*-tests, and at least ordinal in the case of the Mann–Whitney *U*-test.

The results from these three different analyses allow comparisons to be made between groups and Items with respect to different sets of assumptions, balancing the strengths and weaknesses of each. Assessment scores between groups were compared using both an independent *t*-test and Mann–Whitney *U*-test for the same reasons.

## Results

A total of 23 students participated in the study representing the entire cohort of Year 3 students including 17 females and 6 males. Group 1 had twelve participants, while group 2 had eleven participants.

### Perceptions of training on virtual reality haptic simulator

**Fig. 3:**
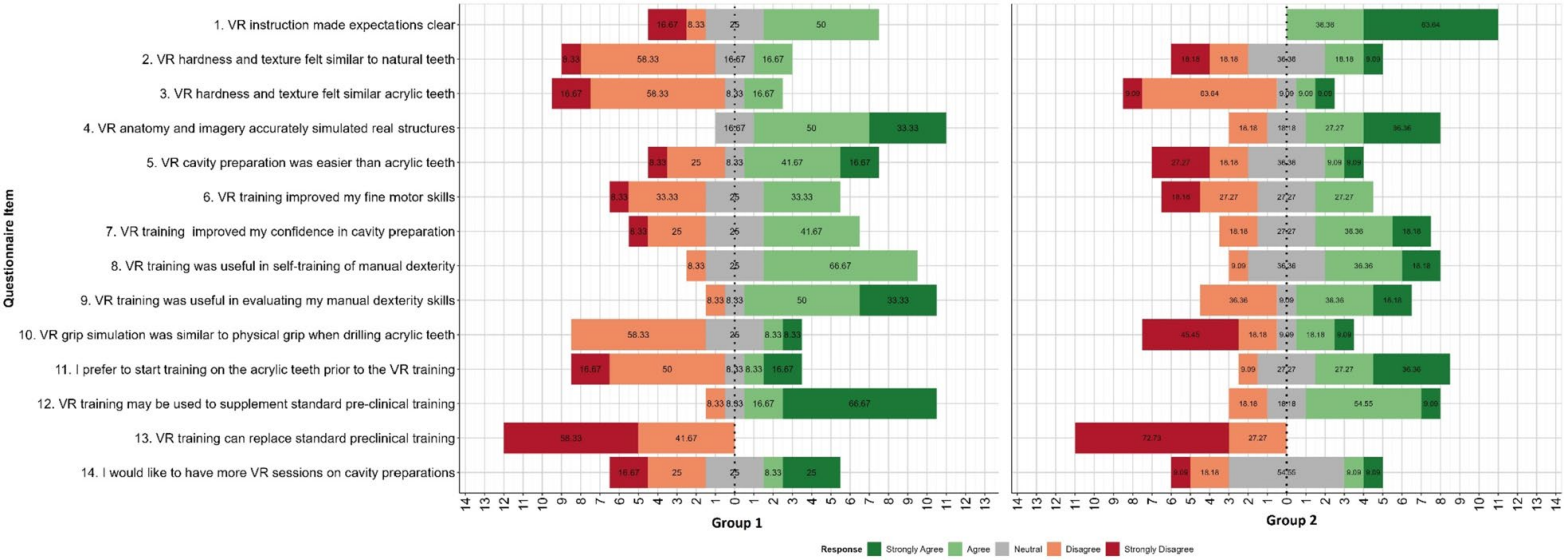
Group 1 agreement responses for each item. Values on bars are percentage of group 1 participants

The agreement responses for each Item, by group, are depicted in Fig. 3.

The responses to each item by group are detailed, along with mean agreement scores for each item for each group when the agreement responses are converted to numerical scores, and which items for which the two groups have statistically different agreement patterns and scores.

These patterns of agreement suggest that, largely, participants in both groups had similar perceptions of VR and agreed on similar patterns to each of the items. The only statistically significant variation was seen for items 1, 11, and 12; respectively, that instruction of VR made the expectations of the task clear (some disagreement from Group 1, M = 0.08, SD = 1.16; full agreement from Group 2, M = 1.64, SD = 0.50), a preference for starting training on acrylic teeth prior to using VR (Group 1 mostly disagreed, M = -0.42, SD = 1.38; Group 2 mostly agreed, M = 0.91, SD = 1.04), and that VR training might be used to supplement standard pre-clinical training (higher levels of Strongly Agree in Group 1, M = 1.42, SD = 1.00, than Group 2, M = 0.55, SD = 0.93, but both groups largely agree to some extent overall).

Agreement response types for each Item, displayed as counts (and percentages) within Group, along with significance test for difference between Group 1 and Group 2 responses, are presented in Table 2. Chi Squared tests (X^2^) of association reflect relationships between Group and agreement distribution, *t*-Tests (t) and Mann Whitney *U*-tests (U) are conducted comparing mean (M) agreement between groups after converting to scores (with scores anchored at Strongly Disagree = -2, Strongly Agree = 2). Standard deviations (SD) for agreement scores are also shown as a measure of variation within each item.

**Table 2:** Agreement response types for each item, displayed as counts and percentages (within group), along with significance test for difference between group 1 and group 2 responses

	**Group 1: VR before Acrylic (*n* = 12)**							**Group 2: VR after Acrylic (*n* = 11)**							**Significance Tests**		
**Item**	*Strongly Disagree*	*Disagree*	*Neutral*	*Agree*	*Strongly Agree*	*M*	*SD*	*Strongly Disagree*	*Disagree*	*Neutral*	*Agree*	*Strongly Agree*	*M*	*SD*	*X*^*2*^	*t*	*U*
1. VR instruction made expectations clear	2 (16.67)	1 (8.33)	3 (25.00)	6 (50.00)	0 (0)	0.08	1.16	0 (0)	0 (0)	0 (0)	4 (36.36)	7 (63.64)	1.64	0.50	0.002	0.001	< 0.001
2. VR hardness and texture felt similar to natural teeth	1 (8.33)	7 (58.33)	2 (16.67)	2 (16.67)	0 (0)	-0.58	0.90	2 (18.18)	2 (18.18)	4 (36.36)	2 (18.18)	1 (9.09)	-0.18	1.25	0.405	0.392	0.385
3. VR hardness and texture felt similar acrylic teeth	2 (16.67)	7 (58.33)	1 (8.33)	2 (16.67)	0 (0)	-0.75	0.97	1 (9.09)	7 (63.64)	1 (9.09)	1 (9.09)	1 (9.09)	-0.55	1.13	> 0.999	0.647	0.700
4. VR anatomy and imagery accurately simulated real structures	0 (0)	0 (0)	2 (16.67)	6 (50.00)	4 (33.33)	1.17	0.72	0 (0)	2 (18.18)	2 (18.18)	3 (27.27)	4 (36.36)	0.82	1.17	0.519	0.406	0.580
5. VR cavity preparation was easier than acrylic teeth	1 (8.33)	3 (25.00)	1 (8.33)	5 (41.67)	2 (16.67)	0.33	1.30	3 (27.27)	2 (18.18)	4 (36.36)	1 (9.09)	1 (9.09)	-0.45	1.29	0.249	0.161	0.157
6. VR training improved my fine motor skills	1 (8.33)	4 (33.33)	3 (25)	4 (33.33)	0 (0)	-0.17	1.03	2 (18.18)	3 (27.27)	3 (27.27)	3 (27.27)	0 (0)	-0.36	1.12	> 0.999	0.666	0.701
7. VR training improved my confidence in cavity preparation	1 (8.33)	3 (25.00)	3 (25.00)	5 (41.67)	0 (0)	0.00	1.04	0 (0)	2 (18.18)	3 (27.27)	4 (36.36)	2 (18.18)	0.55	1.04	0.677	0.223	0.274
8. VR training was useful in self-training of manual dexterity	0 (0)	1 (8.33)	3 (25.00)	8 (66.67)	0 (0)	0.58	0.67	0 (0)	1 (9.09)	4 (36.36)	4 (36.36)	2 (18.18)	0.64	0.92	0.417	0.877	> 0.999
9. VR training was useful in evaluating my manual dexterity skills	0 (0)	1 (8.33)	1 (8.33)	6 (50.00)	4 (33.33)	1.08	0.9	0 (0)	4 (36.36)	1 (9.09)	4 (36.36)	2 (18.18)	0.36	1.21	0.523	0.124	0.151
10. VR grip simulation was similar physical grip when drilling acrylic teeth	0 (0)	7 (58.33)	3 (25.00)	1 (8.33)	1 (8.33)	-0.33	0.98	5 (45.45)	2 (18.18)	1 (9.09)	2 (18.18)	1 (9.09)	-0.73	1.49	0.051	0.469	0.262
11. I prefer to start training on the acrylic teeth prior to the VR training	2 (16.67)	6 (50.00)	1 (8.33)	1 (8.33)	2 (16.67)	-0.42	1.38	0 (0)	1 (9.09)	3 (27.27)	3 (27.27)	4 (36.36)	0.91	1.04	0.081	0.017	0.021
12. VR training may be used to supplement standard pre-clinical training	0 (0)	1 (8.33)	1 (8.33)	2 (16.67)	8 (66.67)	1.42	1.00	0 (0)	2 (18.18)	2 (18.18)	6 (54.55)	1 (9.09)	0.55	0.93	0.037	0.042	0.021
13. VR training can replace standard pre-clinical training	7 (58.33)	5 (41.67)	0 (0)	0 (0)	0 (0)	-1.58	0.51	8 (72.73)	3 (27.27)	0 (0)	0 (0)	0 (0)	-1.73	0.47	0.670	0.490	0.502
14. I would like to have more VR sessions on cavity preparations	2 (16.67)	3 (25.00)	3 (25.00)	1 (8.33)	3 (25.00)	0.00	1.48	1 (9.09)	2 (18.18)	6 (54.55)	1 (9.09)	1 (9.09)	-0.09	1.04	0.777	0.866	> 0.999

*M* mean, *SD* standard deviation, *X*^*2*^ Chi Squared tests, *t t*-Tests, *U* Mann Whitney *U*-tests

General themes emerging from responses to open-ended questions were drawn. Main patterns related to student perceptions are displayed in Table 3.

**Table 3:** Main themes emerging from responses to open-ended questions

Benefits/ enablers of VRHS	Limitations/ barriers to of VRHS	Recommendations for improvement
Safe environment for beginners	Unrealistic sensation/ hardness of teeth compared to natural teeth, not for transition to clinics	Dedicated time for VRHS sessions
Multiple attempts possible, unlike irreversible damage in acrylic teeth	Dissimilar characteristic of VRHS handpiece to real handpiece in terms of grip, weight, and water spray	More practice on extracted natural teeth
Perceived increase in confidence initially	Perceived increase in mental stress, cannot replace conventional dental mannequin experience	Improve handpiece grip and produce simulated water spray
Exposure to/visualization of caries in fissures	Unrealistic maneuver range of head and jaw, dissimilar to real patient	Open/unlimited access to VRHS
Ability to differentiate between tooth layers (enamel, dentin and pulp)	Impractical use of instruments such as mirror and burs	Ability to restore teeth

## Discussion

Pre-clinical teaching of restorative dentistry, specifically operative dentistry, aims at developing manual dexterity, which is fundamental for developing skills and competence in operative techniques across the board. Manual dexterity necessitates the development and consolidation of innate psychomotor skills and also requires simultaneous hand–eye coordination. The main objectives of contemporary operative dentistry are diagnosis of dental caries, removal of diseased tooth structure, cavity preparations based on minimal invasive approaches, and to restore structure, function and aesthetics [21].

In order to achieve the aforementioned objectives, and ensure student competency, the right tools must be provided to mimic real clinical cases and provide a smooth transition to clinics, with the ultimate goal of patient safety and satisfaction. VRHS are currently used as pedagogical tools for training students in the pre-clinical stage [22, 23]. They have evolved extensively over the last decade, being considered “ergonomically accurate” by educators, demonstrating its reliability and capacity to provide a learning environment that enhances student learning and practice experiences [24, 25]. Despite reported advantages of VRHS, conventional mannequin-based simulators remain the most frequently used training tool for restorative dentistry in the pre-clinical environment [26, 27].

Training dental students on restorative procedures using extracted natural teeth are considered the best option as they provide students the appropriate feedback sensation when drilling into the various layers of teeth. However, as there is a general tendency towards restoring and retaining teeth nowadays, having a suitable pool and stable supply of extracted human teeth proves challenging. Also, standardization across large cohorts, and difficulty to simulate occlusal and proximal relations may pose an issue [28, 29]. Acrylic teeth can be used to provide standardized assessments. However, virgin teeth are often used to assess caries removal and cavity preparation, but such tasks remain confined to a shape-cutting exercise rather than a clinically driven exercise [30]. Moreover, they do not accurately simulate the hardness, texture and tactile feedback experienced during cavity preparation on natural teeth. On the other hand, virtual teeth are produced by scanning extracted human teeth, thus presenting a more realistic appearance. In addition, caries can be simulated with a texture as close to real life as possible [29]. In the current study, novice students unsurprisingly preferred practicing on natural teeth as they felt the hardness and texture would be similar to what they would encounter in a patient’s mouth. Participants in both groups had similar perceptions of VRHS. This is in line with the findings of Dwisaptarini et al., (2018), concluding that training on the VRHS had equivalent effects to extracted teeth in improving minimally invasive caries removal [27]. Another study concluded that improvement of the overall cavity preparation scores post-haptic use in short-term was not statistically significant compared to typodont teeth [28]. A recent study by San Diego et al., (2022), reported that equal benefit can be observed with the removal of artificial carious lesions when students were trained by either VRHS or traditional simulations [29]. Bakr et al., (2013), found no clear evidence that early exposure to haptic feedback could better assist in the development of psychomotor skills in restorative dentistry [30]. In contrast, other studies showed improvement in manual dexterity skills with the use of VRHS [31–33]. This could be attributed to the differences in the VRHS device used, the number of participants and the method of evaluating student perceptions. A recent study surveying twenty-seven dental schools, including ones using the same device used in the current study (SIMtoCARE Dente®) found that cariology (92.6%) and manual dexterity (85.2%) were the most employed application by dental trainers within the pre-clinical training [34].

A study by Ria et al., (2018) in a UK dental school assessed the learning progression of first-year dental students using VRHS [35]. Five tasks were assigned to students, two to three weeks apart involving the need to remove caries with increasing difficulty. Improvements in fine motor skills with a more precise conservative approach to caries removal were reported. Another study evaluating the efficacy of VRHS for caries removal with repetitive training over three sessions found a significant improvement after the second session. However, improvements plateaued following the third session with no significant difference between the second and third sessions [36]. Interestingly, in the current study, not all students felt that VRHS improved their fine motor skills. This might be owing to the single session involving one procedure on VRHS, which provided limited practice to appreciate any improvements in their motor skills during caries removal.

Feedback provided to the learner is considered one of the most central variables to effective motor-skills learning. According to Van de Ridder et al., (2008), the goal of feedback in medical education is to improve the trainee’s performance [37]. Debriefing has been described as the ‘‘heart and soul’’ of simulator-based training, providing a better chance to find out the “why” of the actions observed during the simulation exercise, enhancing self-correction and self-assessment [38, 39]. Feedback can be given at the simulator during or after a session, especially when teaching technical or psychomotor skills. Evidence from literature shows that concurrent feedback reinforces psychomotor skills, clarifying the underlying processes expected to reach the outcome, placing the trainee on the right path instantly and decreasing memory demands [40–42]. This is in accordance with findings of the present study, in which more students in group 1 (study group) agreed that VRHS was useful in self-training and evaluation of their motor skills compared to group 2 (control group). This might be due to the fact that group 1 attempted the VRHS first and had the opportunity to self-train, utilizing the feature of multiple attempts on the same tooth.

A variation was observed in the perceptions in the two groups in relation to the sequence of tasks. Group 1 mostly disagreed on starting the training on acrylic teeth prior to using VRHS, while the majority of Group 2 participants reported a preference starting with acrylic teeth first. Responses to open-ended questions also showed that the participants reported an unrealistic maneuver range of VRHS head and jaw, which is different to the experience on the mannequin. The characteristic of VRHS handpiece was different to the physical handpiece in terms of grip, weight, and water spray. All the former reasons suggest that despite some research findings advocating the integration of VRHS early in the undergraduate dental curriculum [19, 43], educators need to be mindful of the sequence, and possibly scheduling the first VRHS session after the introduction of the conventional phantom head laboratory experience, to allow novice students to adjust to the environment, correct seating position and maneuver around the patient, correct use of restorative instruments and presence of adjacent and opposing teeth. Previous studies inferred that in order to achieve significant enhancement in performance, more time should be invested in training students on VRHS, with some reporting a minimum of 3 to 8 h [14, 16, 19, 36]. Students enrolling on dental programs have a strong inclination towards performing hands-on practical tasks as a dentist, and developing a professional status, therefore, it is paramount to enhance these skills in a well-structured manner [44, 45].

According to the qualitative data in the current study, the majority of students in both groups did not consider that VRHS training has the potential to replace conventional dental mannequin training in a pre-clinical laboratory setting. However, VRHS was perceived to be appropriate to supplement pre-clinical training on mannequins. These findings are in consensus with other studies endorsing the use of VRHS as an adjunct rather than an alternative to conventional phantom head simulators [14, 18, 19, 43].

All year 3 students willingly participated in the current study; however, the relatively small number of participants and the single operative procedure might be a limitation. Longitudinal data on how VRHS training translates into clinical competence and empirically determine optimal pedagogical experiences can be further researched.

While refraining from over generalization based on the study results, the following recommendations can be drawn when integrating VRHS into the dental training:

Curriculum design:

- Consideration of the sequence of sessions between VRHS and CSE.
- Triangulation of self, instructor, and device feedback with an ultimate goal of enhancing student performance.
- Training instructors/supervisors to deliver standardized structured feedback tailored to VRHS tasks.

Virtual Reality Haptic Simulator device:

- Further advancements in the VRHS handpiece grip, possibly simulating water spray/splatter.
- Further advancements in the VRHS software, rendering the tactile fidelity more realistic compared to natural teeth.
- Optimizing the viewing screen size, to enhance the user maneuver range around the screen.

## Conclusion

Novice dental students generally perceived VRHS as a useful tool for enhancing their manual dexterity. Findings suggest that training on the VRHS should be employed to support rather than replace conventional mannequin simulated training in pre-clinical restorative dentistry. Dental Institutions should endorse virtual reality technology with caution, ensuring a planned integration into the curriculum to optimize benefit. Feedback is pivotal to effective learning in simulation-based education, and the triangulation of feedback could serve as a powerful aid to maximize the learning experience in a pre-clinical setting. Advancements to the VRHS hardware and software are required to bridge the gap and provide a smooth transition to clinics. Further research into virtual simulation-based teaching and learning pedagogies, with longitudinal data may be indicated.

## Data Availability

The data that supports the findings of this study are available on request from the corresponding author. The data are not publicly available due to ethical restrictions.
